# The impact of impression coping geometrical design on accuracy of implant impressions: an experimental study

**DOI:** 10.1186/s40729-020-00256-0

**Published:** 2020-10-10

**Authors:** Motaz Osman, Neamat Hassan Abubakr, Ahmed Suliman, Hassan Ziada

**Affiliations:** 1 Department of Oral Rehabilitation, Faculty of Dentistry, University of Khartoum, Khartoum, Sudan; 2 Biomedical Sciences, School of Dental Medicine, University of Nevada, Las Vegas, 1001 Shadow Lane, Suite 20, Las Vegas, NV 89106 USA; 3 Department of Oral Maxillofacial Surgery, University of Khartoum, Khartoum, Sudan; 4 Clinical Sciences, School of Dental Medicine, University of Nevada, Las Vegas, Las Vegas, USA

**Keywords:** Implant, Geometrical design, Impression material

## Abstract

**Aim:**

This in vitro study aimed to evaluate the effect of implant impression coping geometrical designs on the accuracy of open and closed impression techniques and in the parallel and nonparallel implant positions.

**Material and methods:**

Three custom-made acrylic resin models of three tested implant systems (Straumann®, SIC Invent®, and Osstem®) with diverse coping geometrical designs were evaluated in simulated cases of two parallel and two nonparallel implants. The horizontal and vertical discrepancies were measured and analyzed.

**Results:**

No statistically significant differences between the two impression techniques in either parallel or nonparallel implants were observed. The high retentive design of the Osstem system showed a statistically significant difference.

**Conclusion:**

The geometrical design of the impression copings did not affect the accuracy for either the open and closed tray techniques. However, the high retentive coping design of the Osstem implant affected the accuracy in the open tray technique.

## Introduction

In implant prosthodontics, an accurate impression is critical in constructing a precise prosthesis. The accuracy may be influenced by the impression material selected as well as the technique, coping design, shape, type of impression tray, implant numbers, implant angulations, and the operator’s skill [1–3]. Other influences include the direction of removal of the tray in relation to the implants’ axis, the number and parallelism of the implants, the degree of undercuts present, and the depth of implant position [4].

There are variations in implant impression coping shapes and designs, depending on the implant system and the components designed by the manufacturer of a particular system [4]. Therefore, to produce an accurate impression, familiarity with coping designs and geometry is required [2, 5, 6].

Several modifications have been proposed to enhance the retention of impression copings. They may be modified by treatment with airborne-particle abrasion or impression adhesives. In 2000, Vigolo et al. found improved precision of the impression when adhesive-coated copings were used [7]. However, Liou and colleagues showed that surface treatment of copings did not lead to increased accuracy [8].

Furthermore, in 2004, Vigolo et al. evaluated the accuracy of three impression coping designs and found that casts retrieved from transfer impressions with nonmodified copings and those with airborne-particle abraded adhesive-coated copings were statistically less accurate than casts from square impression copings splinted with autopolymerizing acrylic resin [9].

It is postulated that the more retentive element of a square impression coping could lead to better entrapment of the impression material, resulting in less discrepancy [6, 9, 10]. In one study, the modified squared and index techniques generated more accurate casts than the squared techniques [11]. Other studies confirmed that the shape and design of the impression coping affect impression accuracy more than the impression technique [5, 12].

The present study hypothesizes that the highly retentive coping design for the open tray technique and the triangular cross-sectional design for the closed tray technique should produce more accurate impressions than the medium and low retentive designs and the rectangular and round cross-sectional designs in the simulated clinical scenario of parallel and nonparallel implants. This study’s clinical relevance is the identification and analysis of the influences of impression coping geometry on accuracy, which is vital in the long-term success of implant-supported prostheses.

This study aimed to evaluate the effect of various impression coping geometrical designs on accuracy, in parallel and nonparallel implants case scenarios, using open and closed implant impression techniques.

## Materials and methods

**Fig. 1:**
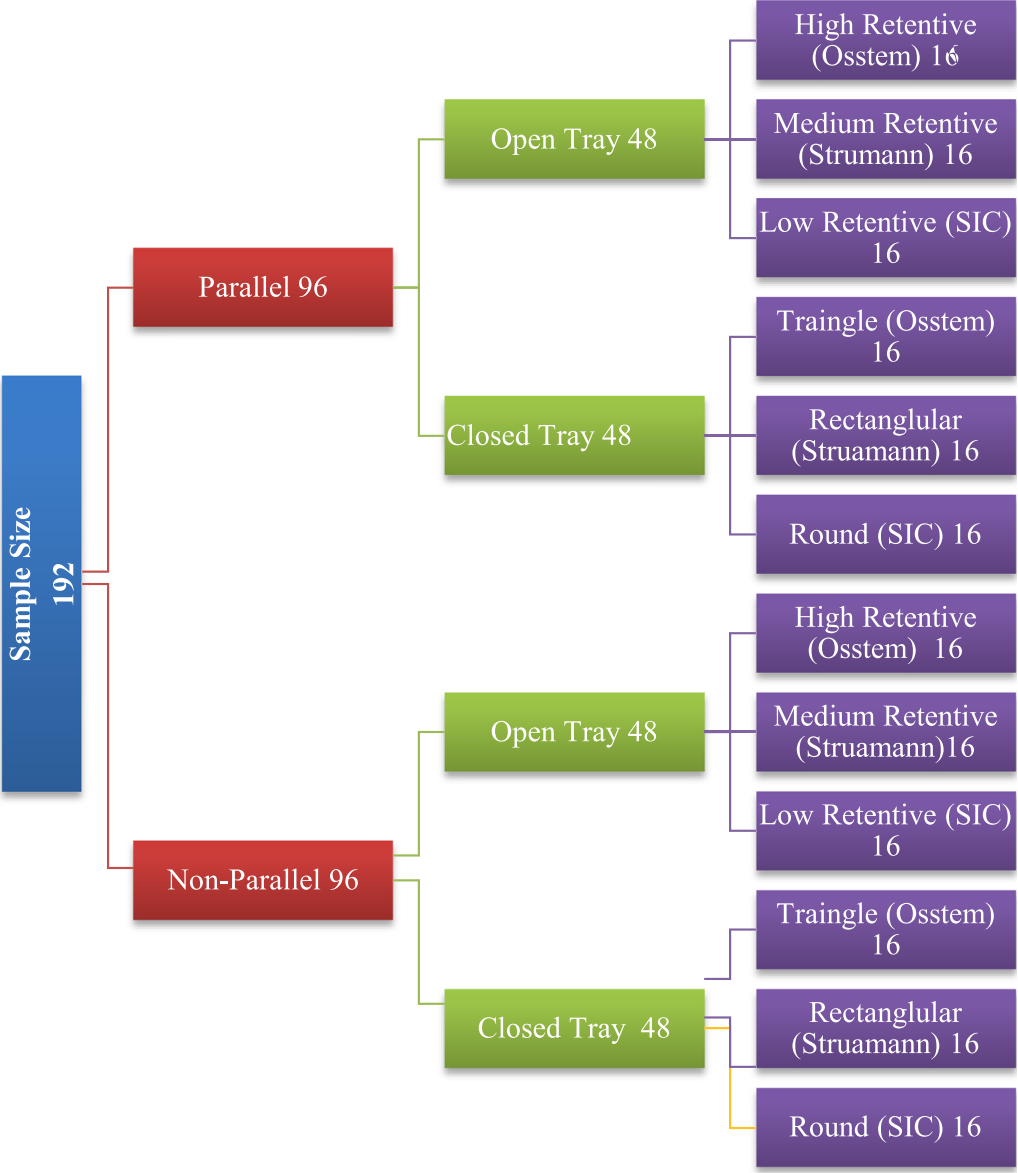
Sample distribution flowchart

The present experimental investigation evaluated the effect of implant impression coping geometrical design on the accuracy in the open and closed implant impression techniques of two simulated case scenarios of parallel and nonparallel implants in a Kennedy class III partially edentulous maxilla (Fig. 1).

### Test model fabrication

Three custom-made acrylic resin models (each model for an implant system) were made from heat-cure acrylic resin (Lucitone-199 DENTSPLY). Each simulated a partially edentulous maxillary Kennedy class III case.

The test models were constructed using the following steps: stone cast simulated a partially edentulous maxillary Kennedy class III including bilaterally missed first premolar, second premolar, and first molar being obtained. Then, silicon impressions were taken for this cast and poured in wax to form three wax modes. These wax models were dewaxed and processed using heat-cured acrylic resin (Lucitone-199 DENTSPLY) to form the three acrylic casts which were used as the test models [13, 14].

For the purpose of standardization of implant insertion and positioning in all the three-testing model, surgical guides were designed using CAM software and produced through 3D printing (Formlabs Form 2 U.S).

Implants were inserted in each test model using a dental surveyor and milling machine (BEGO Paraskop M Germany). Tripoding was performed to ensure reproducible positioning of the test models on the surveyor; one dot was palatal to the incisor teeth in the midline of the model, and two dots were palatal to the area of the second molars, one on the right side and one on the left side. Four implants were installed in each test model; on the right side, the implant in the first premolar area (14) was installed with a long straight axis, and the implant in the first molar (16) was tilted distally and adjusted to 15°. On the left side, the two implants were installed parallel to each other in the areas of the first premolar (24) and first molars (26) [15, 16].

Each test model was then adjusted at 0° and perpendicular to the table of the milling machine. The surgical guide was positioned securely on the test model. Implant drilling was made through the surgical guide sleeve using the sequence of the implant system. Then, the implants were placed and secured, and to ensure implant stability within the model, the inserted implants were cemented using lutting adhesive resin cement (Multilink/Ivoclar Vivadent) [17].

**Fig. 2:**
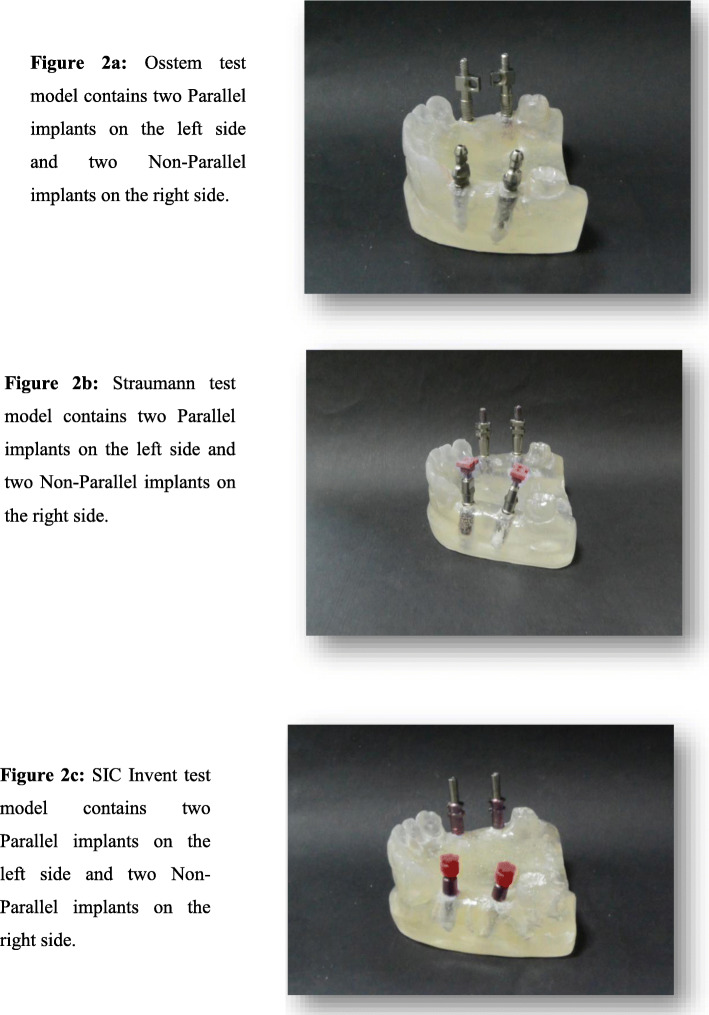
**a** Osstem test model with two parallel implants on the left side and two nonparallel implants on the right side. **b** Straumann test model with two parallel implants on the left side and two nonparallel implants on the right side. **c** SIC Invent test model with two parallel implants on the left side and two nonparallel implants on the right side.

Three test models were obtained (Fig. 2a–c):

First test model (2a): Osstem Implant System (Seoul, Korea) with an implant fixture diameter of 4.0 mm and length of 11.5 mm.

Second test model (2b): Straumann Implant System (AG) with an implant fixture diameter 4.1 mm and length: 10.0 mm.

Third test model (2c): SIC Invent Implant System (SIC Invent Deutschland GmbH, Germany) with an implant fixture diameter of 4.0 mm and length of 11.5 mm.

The impression copings are supplied in different designs and shapes; besides connection, it has various length, width, indentation depth, and structure. The external geometrical design of the impression copings may consist of various length of projection and their multiplications or another design of different shapes like square, rectangular, triangle, and round or sometimes combinations of these features. Also, modification of the coping surface such as airborne-particle abrasions or copings coats with the compatible adhesive could enhance the accuracy of the impressions. The impression copings of the three implant systems are classified according to their geometrical designs as follows:

I- For the open tray impression technique, they are classified according to the external projection into high retentive (Osstem), medium retentive (Straumann), and low retentive (SIC Invent).
II- For the closed tray impression technique, they are classified according to external shape into triangular cross section (Osstem), rectangular cross section (Straumann), and round cross section (SIC Invent) [5, 9, 12].

The three retentive designs of the copings of the open tray technique were evaluated (high retentive for the Osstem, medium for the Straumann system, and low retentive for the SIC Invent system). For the closed tray technique, the triangular cross section of the Osstem system, rectangular cross section of the Straumann system, and the round cross section of the SIC Invent system were evaluated.

For the open tray fabrication, the impression copings of the open tray were secured into their corresponding implant analogs. A sheet of wax (Bego/Germany) was adapted over the palatal area of the maxillary region, and then another two sheets of wax were adapted around the impression copings in all directions (mesial, distal. labial, and palatal). Square sections of wax were then removed to form stoppers. These stoppers were made to ensure even thickness of the impression material and to ensure the same allocation and pressure as the impression tray. The self-cured acrylic resin (Lucitone Fas-Por+/DENTSPLY) was mixed according to the manufacturer’s instructions and adapted over the wax spacer. After complete setting, a handle was made, and finishing and perforations were made on the tray [18].

The calculated sample size was established using data from a previous study [19]. A sample size of 13 for each technique was calculated based on a power calculation for analysis. This number was increased to 16 to allow for potential errors while conducting the study. The overall sample size was 192 impressions (96 parallel and 96 nonparallel implants). In the parallel group, 48 impressions were made for each implant impression technique (open and closed) for the three test models. Similarly, for the nonparallel group, 48 impressions for each implant impression technique (open and closed) were made for the three test models (Fig. 1).

The impressions were then made from Virtual Monophase vinyl polysiloxane (Ivoclar Vivadent AG), and their accuracy was evaluated using the criteria described by Lee and Gallucci [20]:

There should be an exact imprint and reproduction of the implant areas.

The impression copings should not be displaced from the impression.

There should be no voids in the occlusal, buccal, lingual, or interproximal surfaces of the neighboring teeth.

The impression material should not be separated from the custom tray.

Any impression that did not meet these acceptance criteria was repeated until the criteria were met. Furthermore, to eliminate the potential of repositioning failures/error, two examiners were involved in the evaluations (interexaminer reliability of 0.932), and any impressions not meeting the proper repositioning in the closed tray were repeated until the impression was deemed satisfactory.

The horizontal discrepancy between the coping for the test and the master casts was evaluated and recorded for analysis. The measurements were recorded and used to compare the horizontal distance measurements between the test models and the cast digital measurements for every technique and each implant system [21].

## Horizontal measurement on the test model

The three models were scanned using a high-resolution reference dental scanner (Activity 885 Smart Optics - Sensortechnik GmbH, Bochum, Germany) with ratio accuracy according to DIN-ISO-12836-8 μm. To avoid glossy surface reflections, a single layer of powder (SHERA SCAN SPRAY-Germany) was applied on the surface of the test models before scanning. The horizontal distances between the two implants in parallel and non-parallel sides were measured from the center to the center of the implant fixture, using software (exocad-Dental CAD). The distance on the right side between implants 14 (with long axis) and 16 (angulated distally 15 degrees) was assigned as D1, and the distance on the left side between implants 24 and 26 (parallel implants) was assigned as D2 [21].

## Horizontal measurements on the master cast

For the horizontal distance measurements, the master cast was scanned by an Activity 885 Smart Optics scanner, and D1 and D2 were measured using software (exocad-Dental CAD). These measurements were recorded and then used to compare between the two different impression techniques.

## Study measurements (Fig. 3)

The impression accuracy was evaluated using the following parameters:

1. The horizontal discrepancy involved the differences in the horizontal distance measurement between the test model and the master cast:
  A) The first horizontal measurement on the test model involved the horizontal distance between A-B (D1) and C-D (D2).
  B) The second horizontal measurement on the master cast involved the horizontal distance between A-B (D1) and C-D (D2).
2. The vertical discrepancy involved the absence or presence of vertical discrepancy that encountered between the verification jig and the laboratory implant analog in the master cast.

**Fig. 3:**
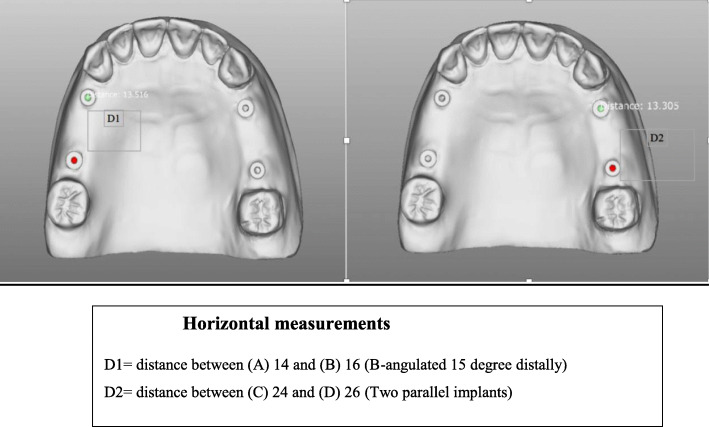
Horizontal measurements

For the vertical discrepancy, the evaluation was a dictum of absence and presence of vertical discrepancy encountered under the stereomicroscope (AmScop®14370, Myford Road, #150, Irvine, CA 92606, USA) at × 50 magnifications and related data recorded. This evaluation was made using verification jigs fabricated from a CAD/CAM acrylic resin block (BILKIM® PMMA blank for CAD-CAM applications 14 mm-A2 color-Turkey). Accordingly, the stone casts were sectioned to a base of 20 mm, to facilitate placement under the stereomicroscope [21].

Data were tabulated and statistically analyzed using IBM SPSS Statistics software version 22. The information collected from the three implant systems was classified and used to compare the effect of implant impression techniques and parallelism. The *P* value was set at *P* ≤ 0.05 and regarded to be statistically significant.

Where data were not normally distributed, the nonparametric test, the Mann-Whitney *U* test, and Kruskal-Wallis test were used. The median and interquartile range (IQR) were used as a measure of the central tendency to the data. The *P* value was also set at *P* ≤ 0.05 and regarded to be statistically significant.

## Results

Three different impression coping designs were evaluated for the open tray technique, which were the high retentive Osstem coping, the medium retentive Straumann coping, and the low retentive SIC Invent coping, for the open tray techniques. For the closed tray technique, the impression coping designs were the triangular coping of the Osstem implant, the rectangular coping of Straumann implant, and the round coping of the SIC Invent implant.

For the horizontal measurement, the data were not normally distributed; hence, the Mann-Whitney *U* test and the Kruskal-Wallis test were used.

The Kruskal-Wallis test in Table 1 shows no statistically significant differences in impression accuracy in the horizontal direction between the open tray coping designs (*P* value 0.068). Similarly, there were no statistically significant differences in impression accuracy for the horizontal measurements between the geometrical designs in the closed tray technique (*P* value 0.654) (Table 1). The lowest interquartile range (IQR) observed was for the high retentive impression coping of the Osstem (0.009) in the open tray technique, while for the closed tray technique, the rectangular design of the Straumann showed the lowest IQR (0.023).

**Table 1:** Horizontal measurement in the open and closed tray impression techniques

Impression techniques		*N*	Median	IQR	*P* value
Open tray	**High retentive (Osstem)**	32	0.011	0.009	**0.068**
	**Medium retentive (Straumann)**	32	0.015	0.014	
	**Low retentive (SIC)**	32	0.020	0.029	
Closed tray	**Triangular (Osstem)**	32	0.037	0.056	**0.654**
	**Rectangular (Straumann)**	32	0.016	0.023	
	**Round SIC**	32	0.036	0.036	

Kruskal-Wallis test, *P* value > 0.05 no significance difference

For the parallel and nonparallel implants (implant angulation) in the open tray technique, the Kruskal-Wallis test also showed no statistically significant differences (*P* value 0.059, 0.0852 respectively) in the horizontal measurements between the three coping geometrical designs (Table 2). For the parallel and nonparallel implant, the lowest interquartile range (IQR) observed was for the high retentive impression coping of the Osstem implant (0.005 and 0.010 respectively).

**Table 2:** Effect of implant angulation on the accuracy in the open tray coping design in the horizontal measurements

Angulations	Coping design	*N*	Median	IQR	*P* value
Parallel implant	**High retentive (Osstem)**	16	0.008	0.005	**0.059**
	**Medium retentive (Straumann)**	16	0.017	0.010	
	**Low retentive (SIC)**	16	0.019	0.030	
Non-parallel implant	**High retentive (Osstem)**	16	0.016	0.010	**0.852**
	**Medium retentive (Straumann)**	16	0.016	0.026	
	**Low retentive (SIC)**	16	0.020	0.029	

Kruskal-Wallis test, *P* value > 0.05 no significance difference

The Kruskal-Wallis test presented in Table 3 also shows no statistically significant differences for the closed tray coping geometrical designs, in the horizontal measurements, and in the parallel and nonparallel implants (*P* values 0.576 and 0.908, respectively). For the parallel and nonparallel implants, the lowest interquartile range (IQR) observed was for the rectangular design impression coping of the Straumann (0.024 and 0.022 respectively).

**Table 3:** Effect of implant angulation on accuracy in the closed tray impression coping design in the horizontal measurements

Angulation	Coping design	*N*	Median	IQR	*P* value
Parallel implant	**Triangular (Osstem)**	16	0.026	0.042	**0.576**
	**Rectangular (Straumann)**	16	0.014	0.024	
	**Round (SIC)**	16	0.028	0.032	
Non-parallel implant	**Triangular (Osstem)**	16	0.039	0.076	**0.908**
	**Rectangular (Straumann)**	16	0.016	0.022	
	**Round (SIC)**	16	0.041	0.036	

Kruskal-Wallis test, *P* value >0.05 no significance difference

The Mann-Whitney test presented in Table 4 compares the effect of implant angulation for the various impression coping designs in the horizontal measurements. The only statistically significant difference found was in the high retentive coping design of the Osstem implant (*P* value 0.0166*). For the open tray, the lowest interquartile range (IQR) observed was for the high retentive design impression) coping of the Osstem system for both the parallel implants (0.005) and the nonparallel implants (0.010). For the closed tray technique, the lowest IQR was for the rectangular design impression coping of the Straumann system for both the parallel implants (0.024) and the nonparallel implants (0.022).

**Table 4:** Effect of impression coping design and implant angulation on accuracy

Impression technique	Coping design	Angulation	Count	Median	IQR	*P* value
Open tray	**High retentive (Osstem)**	**Parallel**	16	0.008	0.005	0.0166*
		**Non-parallel**	16	0.016	0.010	
	**Medium retentive (Straumann)**	**Parallel**	16	0.017	0.010	**0.926**
		**Non-parallel**	16	0.015	0.026	
	**Low retentive (SIC)**	**Parallel**	16	0.019	0.030	**0.999**
		**Non-parallel**	16	0.020	0.029	
Closed tray	**Triangular (Osstem)**	**Parallel**	16	0.026	0.042	**0.423**
		**Non-parallel**	16	0.039	0.076	
	**Rectangular (Straumann)**	**Parallel**	16	0.015	0.024	**0.196**
		**Non-parallel**	16	0.016	0.022	
	**Round (SIC)**	**Parallel**	16	0.028	0.037	**0.616**
		**Non-parallel**	16	0.041	0.036	

*Mann-Whitney test, *P* value < 0.05 statistically significant

For the vertical discrepancy, which was a dictum of either present or absent, the chi-square test showed no statistically significant differences between the open tray coping designs (*P* value 0.440). Similarly, as shown in Table 5, no statistically significant differences were found between coping designs of the closed tray (*P* value 0.720).

**Table 5:** Effect of impression coping design on vertical discrepancy

Impression technique	Coping design	Vertical discrepancy				Total	*P* value
		Yes	Percent %	No	Percent %		
Open tray	**High retentive (Osstem)**	5	15.6	27	84.3	32	**0.440**
	**Medium retentive (Straumann)**	6	18.8	26	81.2	32	
	**Low retentive (SIC)**	9	28.1	23	71.9	32	
	**Total**	**20**	**22.9**	**76**	**79.1**	**96**	
Closed tray	**Triangular (Osstem)**	11	34.4	21	65.6	32	**0.720**
	**Rectangular (Straumann)**	9	28.1	23	71.9	32	
	**Round SIC**	12	37.5	20	62.5	32	
	**Total**	**32**	**33.3**	**64**	**66.7**	**96**	

Chi-square test, *P* value > 0.05 no significance difference

For the parallel implants and the non-parallel implants, the chi-square results in Table 6 revealed no statistically significant differences in the vertical discrepancy between the open tray coping designs (*P* value 0.549 and 0.717, respectively).

**Table 6:** Effect of implant angulation of the open tray coping designs on vertical discrepancy

Angulation	Coping design	Vertical discrepancy				Total	*P* value
		Yes	Percent %	No	Percent %		
Parallel implant	**High retentive (Osstem)**	2	12.5	14	87.5	16	**0.549**
	**Medium retentive (Straumann)**	2	12.5	14	87.5	16	
	**Low retentive (SIC)**	4	25.0	12	75.0	16	
	**Total**	**8**	**16.7**	**40**	**83.3**	**48**	
Non-parallel implant	**High retentive (Osstem)**	3	18.8	13	81.2	16	**0.717**
	**Medium retentive (Straumann)**	4	25.0	12	75.0	16	
	**Low retentive (SIC)**	5	31.3	11	68.7	16	
	**Total**	**12**	**25.0**	**36**	**75.0**	**48**	

Chi-square test, *P* value > 0.05 no significance difference

The chi-square test for the parallel implants showed no statistically significance differences in vertical discrepancies between closed tray coping designs (*P* value 0.904).

Regarding nonparallel implants, similarly, no statistically significant differences between the closed tray coping designs were observed (*P* value 0.766) (Table 7).

**Table 7:** Effect of implant angulation of the closed tray coping designs on the vertical discrepancy

Angulation	Coping design	Vertical discrepancy				Total	*P* value
		Yes	Percent %	No	Percent %		
Parallel implant	**Triangular (Osstem)**	5	31.3	11	68.7	16	**0.904**
	**Rectangular (Straumann)**	4	25.0	12	75.0	16	
	**Round (SIC)**	5	31.3	11	68.7	16	
	**Total**	**14**	29.2	**34**	70.8	**48**	
Non-parallel implant	**Triangular (Osstem)**	6	37.5	10	62.5	16	**0.766**
	**Rectangular (Straumann)**	5	31.3	11	68.7	16	
	**Round (SIC)**	7	43.8	9	56.2	16	
	**Total**	**18**	37.5	**30**	62.5	**48**	

Chi-square test, *P* value > 0.05 no significance difference

## Discussion

Impression copings can have various designs and shapes, connections, lengths, widths, indentation depths, and structures. The horizontal measurement distortion differences between the tested and cast models showed no significant difference between the open and the closed implant impression techniques, in agreement with the findings of Rashidan et al. [5].

The findings of the present study showed that there were no statistically significant differences between coping designs for the open tray (medium retentive Straumann) and closed tray (rectangular Straumann) techniques in agreement with Sabouhi et al. [12].

This study also showed no significant difference between the high retentive coping design of the Osstem implant, the medium retentive design of the Straumann implant, and the low retentive design of the SIC Invent implant. However, the high retentive design Osstem system had slightly better results supporting the suggestion that the more retentive elements of the coping result in more rigidity, the more stability within the impression and consequently, a more accurate master cast [13]. It could also be suggested that the more significant retentive element in the open tray coping increases the contact surface with better engagement inside the impression material. This contact may not only increase the stability but also reduce the impression coping rotational movements. Indeed, Wee in 2000 suggested that one of the requirements for an impression material for the direct implant impression technique is rigidity, holding the impression coping and preventing accidental displacement [22].

Conrad et al. [23] and Kim et al. [24] showed that the process of repositioning impression copings is the leading cause of inaccuracy. However, this issue was not observed in this study, particularly in the closed tray technique.

Some of the present findings were contradictory to those of Rashidan et al. [5]. The authors evaluated the accuracy of impressions techniques using different coping shapes. The shape of the impression coping has been reported to have a greater impact on accuracy than impression techniques [5]. However, the results of the current research demonstrated that the geometrical design of the impression copings did not affect accuracy in either the open and closed tray techniques

In the present study, the observed differences between parallel and nonparallel implants in the horizontal plane were not significant for the high retentive (Osstem), medium retentive elements (Straumann), or low retentive (SIC Invent) element.

The high retentive element exhibited better results than the other designs for both parallel and nonparallel implants, which is evident by the significant differences between parallel and nonparallel implants for the high retentive coping design (Osstem implant system). Jo et al. reported findings similar to those of the current study, even though they had a different methodology [25].

The findings of the present study also do not support the suggestion of Rashidan et al*.* [5] that the more significant vertical distortion occurs in the more retentive coping design, due to higher stresses generated between the impression material and the impression copings during removal from the internal connection implants.

One of the limitations of the current in vitro study was the lack of 3-dimensional analysis, which would have provided more comprehensive evaluation and analysis [26]. Hence, some information may be incomplete due to the use of 2-dimensional evaluation and analysis (horizontal/vertical only). Furthermore, the information and data obtained may vary from those obtained from clinical evaluation, and accordingly, the results of this study should be interpreted and taken with these limitations in mind. Nevertheless, the approach of this study is still considered a perceptive and straightforward means for evaluating the accuracy of the different impression techniques [14, 24]. In addition, other variables such as contraction of the impression material, operator errors, and multiple implants could influence the impression accuracy.

## Conclusion

The geometrical design of the impression copings did not affect the accuracy of the open or closed tray implant impression techniques in the vertical measurements. In the horizontal measurements, the high retentive coping design of the Osstem implant affected the accuracy in the open tray technique.

## Data Availability

All data generated or analyzed during this study data can be independently analyzed.

## Abbreviations

CAM: Computer-aided manufacturing
CAD-CAM or CAD/CAM: Computer-aided design/computer-aided manufacturing
3D: Three dimensional
